# Reactive Oxygen Species in Vascular Formation and Development

**DOI:** 10.1155/2013/374963

**Published:** 2013-01-22

**Authors:** Yijiang Zhou, Hui Yan, Meiqun Guo, Jianhua Zhu, Qingzhong Xiao, Li Zhang

**Affiliations:** ^1^Department of Cardiology, The First Affiliated Hospital, College of Medicine, Zhejiang University, 79 Qingchun Road, Hangzhou, Zhejiang 310003, China; ^2^Centre for Clinical Pharmacology, William Harvey Research Institute, Barts and the London School of Medicine and Dentistry, Queen Mary University of London, London EC1M 6BQ, UK

## Abstract

Reactive oxygen species (ROS) are derived from the metabolism of oxygen and are traditionally viewed as toxic byproducts that cause damage to biomolecules. It is now becoming widely acknowledged that ROS are key modulators in a variety of biological processes and pathological states. ROS mediate key signaling transduction pathways by reversible oxidation of certain signaling components and are involved in the signaling of growth factors, G-protein-coupled receptors, Notch, and Wnt and its downstream cascades including MAPK, JAK-STAT, NF-**κ**B, and PI3K/AKT. Vascular formation and development is one of the most important events during embryogenesis and is vital for postnasal tissue repair. In this paper, we will discuss how ROS regulate different steps in vascular development, including smooth muscle cell differentiation, angiogenesis, endothelial progenitor cells recruitment, and vascular cell migration.

## 1. Introduction

Reactive oxygen species (ROS) are a class of molecules derived from the metabolism of oxygen (O_2_), and are characterized by high chemical reactivity. They include free radicals, such as superoxide (O_2_^−^), superoxide anion radical (O_2_^∙−^), hydroxyl radicals (OH^∙^), and peroxynitrate (ONOO^−^), and nonradicals such as hydrogen peroxide (H_2_O_2_), ozone (O_3_), and hypochlorous acid (HOCl). Traditionally viewed as toxic byproducts of metabolism, ROS cause damage to lipids, membranes, proteins, and DNA through free-radical-mediated chain reaction. Over decades, numerous studies showed that increased oxidative stress plays a central role in the pathogenesis of vascular disease, including hypertension, atherosclerosis, and restenosis. Recent evidence, however, clearly demonstrated that that moderate concentration of ROS acts as intracellular signaling molecules and thereby mediates diverse developmental and physiological processes. 

ROS are important mediators and signal modifiers during various biological processes. Signal transduction mediated by ROS, known as “Redox signaling,” usually involves reversible and oxidation/reduction-based modification of components in signaling pathway. ROS are produced in response to various stimuli, including growth factors, cytokines, chemotactic factors, hypoxia, and shear stress. In turn, many vital biological pathways or cascades are tuned via ROS, such as GPCR, Notch [1] and Wnt-*β*-catenin [2], MAPK, JAK-STAT, NF-*κ*B, and PI3K/AKT. Transcription factors such as HIF1-*α*, AP-1, and NF-*κ*B can themselves be directly modified in a redox-sensitive manner, thereby leading an altered transcriptional profile of gene products. Noteworthy, redox singling is spatially regulated and confined in certain subcellular region. The compartmentalization of redox signaling ensures its specificity in gene regulation and cellular functions, and that ROS can participates in more dynamic cell behaviors that needs different parts of the cell to work together, just as in the case of cell migration.

To fulfill the organism's metabolic demand for oxygen and nutrients, blood vessel formation is one of the earliest and most vital events during embryonic development. Vascular formation are coordinated in a number of steps, which include differentiation and proliferation of endothelial cells (vasculogenesis), blood vessel sprouting and branching (angiogenesis), and differentiation and migration of vascular smooth muscle cells (VSMCs) to cover vessel tube (arteriogenesis) [3, 4]. During these processes, ROS can potentiate angiogenic response by facilitating signaling of multiple angiogenic factors, such as vascular endothelial growth factor (VEGF) and angiopoietin, and enhancing the activity of hypoxia-induced factor (HIF) as well. Vascular repair and regeneration in response to tissue injury or intravascular manipulations also involves angiogenesis. Postnatal neovascularizations are often accompanied by neointimal formation, and these repairing processes involves highly regulated steps, including progenitor cells mobilization and differentiation, vascular cell migration, and VSMCs phenotypic transition, with ROS as an indispensable player. In this paper, we will discuss how ROS regulates various steps of vascular formation and development, including smooth muscle cell differentiation, angiogenesis, endothelial progenitor cells recruitment, and vascular cell migration.

## 2. ROS Source and Nox Enzyme Family

 ROS is generated through a cascade of biological reactions following the formation of superoxide, which can be dismutated to hydrogen peroxide spontaneously or in a reaction catalyzed by superoxide dismutase. Superoxide can also react with nitric oxide to form peroxynitrate. Multiple enzyme systems including nicotinamide adenine dinucleotide phosphate (NADPH) oxidases (Nox) family, xanthine oxidase, mitochondrial respiratory chain, uncoupled eNOS, myeloperoxidase (MPO), lipoxygenase, cyclooxygenase, cytochrome p450, and heme oxygenase have been implicated in the generation of ROS. Among these enzyme systems, the major source of ROS in vascular system, however, is the Noxs.

First characterized in phagocytes like macrophage and neutrophils, the prototypic NADPH oxidase 2 (Nox2, also known as gp91phox) is found to be responsible for antimicrobial defense. With binding to other regulatory subunits, namely, p40phox, p47phox, p67phox, and Rac, the assembled NADPH oxidases complex is able to produce ROS in a “respiratory burst” to kill internalized bacteria. Subsequently, growing biochemical and functional evidence suggests the presence of NADPH oxidase-like activities in nonphagocytic cells, which eventually lead to the discovery of a whole family of NADPH oxidases. The NADPH oxidase family is composed of catalytic subunits termed Nox1-5 and Doux1 and Duox 2, two organizer subunits p47phox and Noxo1, two activator subunits p67phox and Noxa1, and other regulatory subunits like p22phox and p40phox and the binding partner Rac. Different Noxs exhibit tissue-specific distribution and display distinct functions. In vasculature, Nox1, Nox2, Nox4, and Nox5 are mainly expressed. In endothelial cells, Nox2 and Nox4 are responsible for the basal ROS generation [5], but mediate distinctive activation pattern under different stimulation [6]. 

All Nox family members are transmembrane proteins that contain conserved structures: a C-terminal NADPH binding domain, a flavin adenine dinucleotide (FAD) binding domain, six transmembrane domains, and four highly conserved heme-binding histines in the third and fifth transmembrane domains [7]. Based on predicted domain structures, Nox isoforms can be classified into three groups: (1) Nox1-4 display up to 60% homology in amino acid sequence and are predicted to contain six transmembrane *α*-helices and an NADPH-binding domain towards the C-terminus; (2) Nox5 has the same basic structure as Nox1-Nox4 but includes an additional four calcium-binding EF-hand motifs within its N-terminus; (3) Duox1 and Duox2 are similar to Nox5 but include an additional N-terminal peroxidase homology domain on the extracellular site of the membrane.

Superoxide is generated by a complex reaction that takes place once NADPH is bounded to the cytosolic COOH terminus. Electron transfer occurs initially from NADPH to reduce FAD to FADH2. Then single electron transfer occurs through the heme groups, which is then accepted by an oxygen which must be bound to the outer heme group on the opposite side of the membrane. For every one NADPH reduced two superoxide molecules are created.

## 3. ROS and Stem Cell Function Involved in Vascular Formation and Development

### 3.1. Embryonic Stem Cells

Stem cells possess the ability for indefinite self-renewal and potency of differentiation into specialized cell type. The rapidly advancing research field in stem cells, especially with the advent of induced pluripotent stem cells (iPSCs), holds great promise for tissue engineering and regenerative medicine. The fate of stem cells, that is, whether to self-replicate or to differentiate, is tightly regulated by various extracellular cues and intracellular signaling, in which the role of ROS has recently been discovered. 

Embryonic stem cells (ESCs), derived from the inner cell mass of the blastocyst, are pluripotent to generate any cell type from all three primary germ layers. A growing body of evidence suggests that ROS generation and signaling are involved in ESCs differentiation. A single direct current field pulse applied to early embryoid bodies increased intracellular ROS and promotes cardiomyocyte differentiation; this effect can be hampered by free radical scavengers [8]. It was later confirmed NADPH oxidases are vital to drive cardiomyogenesis through MAPK activation and nuclear translocation of cardiac transcription factor myocyte enhancer factor (MEF2C) [9, 10]. Interestingly, mechanical strain-induced cardiovascular differentiation also utilizes Nox-derived ROS family as a signal transducer [11]. Similar evidences of ROS in promoting stem cell differentiation are also demonstrated in many other cell types, including smooth muscle cells [12–14], endothelia cells [15], skeletal muscles [16], neurons [17], adipocyte [18], and chondrocyte [19]. 

### 3.2. Stem Cell Niche and Hematopoietic Stem Cells Maintenance

The stem cell niche, defined as local tissue microenvironment that includes cellular and acellular components, integrates systemic and local cues to regulate stem cell biology [20]. Oxygen tension as a component of metabolic milieu, seems to play a role. Early embryogenesis takes place in female reproductive tract with a low oxygen environment of less than 5%, which seems to be the optimum concentration for mammalian embryonic development [21]. In fact, human ESCs (hESCs) are best-maintained pluripotent under 1–4% oxygen tension with enhanced formation of embryoid bodies and preserved proliferation ability [22]. hESCs began to differentiate spontaneously when culturing under 21% oxygen.

The best-characterized stem cell niche is that of hematopoietic stem cells (HSCs). HSCs with long-term reconstitution activity (LT-HSCs) primarily reside in endosteal zone of bone marrow, where blood perfusion is very limited and oxygen tension can be as low as 1% [23, 24]. Such hypoxic conditions help HSCs maintain slow-cycling proliferation rate and enhanced engraftment ability, while protecting them from potential oxidative stress in more well-oxygenated tissue [20]. HSCs with lower ROS residing in a low-oxygenic osteoblastic niche have a more durable self-renewal activity than those with high ROS [25]. 

Knockout studies provide more evidence of ROS in regulating HSC fate and function. Atm^−/−^ and FoxO1/3/4^−/−^ mice showed defect in HSCs quiescence maintenance and HSCs exhaustion, due, at least partially, to increased ROS level [26, 27]. Treatment with an antioxidant can reextend the HSCs lifespan and restore cell cycle in these deficient cells. Another knockout study found that AKT1/2^−/−^ HSCs retains in quiescence accompanied by lower ROS content, which can be rescued to differentiate after pharmacologically increasing ROS differentiation [28]. This view is also confirmed in *Drosophila*, as ROS prime hematopoietic progenitor for differentiation through activation of FoxO and JNK and downregulation of polycomb [29]. In addition, in cardiac and embryonic stem cells, physiological levels of intracellular ROS are required for maintaining genomic stability through activating the DNA repair pathway [30]. Thus, fine tuning of ROS levels is essential for stem cell function; with sufficient ROS required for differentiation, and low ROS for stemness maintenance and quiescence. 

### 3.3. Endothelial Progenitor Cells

Postnatal neovascularization in ischemic insults is critical for tissue repair, and involves both angiogenesis from preexisting vessels and *de novo *vasculogenesis to form new vessels. There is firm evidence that various stem/progenitor cells are mobilized from bone marrow to participate in the process, in which endothelia progenitor cells (EPCs) received special attention. However, the nomenclature and characterization of EPCs are rather unambiguous, and many cell lineages claimed to contain EPCs actually do not have direct evidence to differentiate into vascular cells [31, 32]. Here we still use the term EPC for convenience to refer to differently labeled endothelial progenitors in different studies. 

As discussed above, an appropriate level of ROS is important for HSCs senescence and differentiation. What is more, hematopoietic progenitors release from bone marrows also depends on ROS signaling, as granulocyte colony-stimulating factor- (G-CSF-) induced mobilization of EPCs (sca-1^+^c-kit^+^Lin^−^cells) and other progenitors are strongly prevented by antioxidant N-acetyl-L-cysteine (NAC), as well as their chemotactic migration to stromal cell-derived factor-1 (SDF-1) [33]. In a hindlimb ischemia model, Nox2 knockout mice display reduction of ischemia-induced flow recovery and impaired EPCs (c-kit^+^Flk1^+^cells) mobilization, both of which can be rescued by transplantation of wild-type bone marrow [34]. Mobilization of Nox2^−/−^ EPCs (sca-1^+^flk-1^+^lin^−^) is also blocked in hypoxia condition or EPO stimulation, due to defective production of ROS to inactivate SHP-2, which normally dephosphorylates and inactivates STAT5 downstream EPO signaling [35]. Moreover, Nox2^−/−^ c-kit^+^Lin^−^ bone marrow stem cells show impaired migration and actin polarization in SDF-1-directed chemotaxis [34].

In bone marrows, matrix degrading and remodeling by protease is important for progenitor cell egress and release of cytokines like VEGF and soluble Kit-ligand (sKitL) [36, 37], which guides activation and chemotactic migration of EPCs. Production of Nox2-derived ROS can be activated by leptin binding to its receptor (ObR) in bone marrow cells [38]. With ROS, matrix metalloproteinase-9 (MMP9) is then upregulated, shedding and releasing sKitL to enhance EPCs (sca-1^+^Flk1^+^ cells) mobilization. In addition, the association of EPCs and targeted vessel may also involve ROS, since ROS-dependent expression of vascular cell adhesion molecule-1 (VCAM-1) expression on endothelial cells can promote efficient recruitment and proliferation of LSKCD34- (Lin^−^Sca-1^+^cKit^+^CD34^−^) hematopoietic cells [39].

## 4. ROS and Endothelial Cell (EC) Function Involved in Vascular Formation

Vasculogenesis and angiogenesis are core events during embryonic development for supply of metabolic substrate. Postnatal form of angiogenesis, named neovascularization, also has significance implications in various pathophysiological states like ischemia, wound healing and cancer progression. Angiogenesis is a fined regulated process involving multiple steps including EPC mobilization and differentiation, EC proliferation and migration, and matrix remodeling, almost all of which are found to be modulated by redox signaling. In fact, Nox2 knockout mice display impaired neovascularization in hind limb ischemia [40] and their ECs have much reduced VEGF-induced proliferation and migration [41]. 

### 4.1. EC Migration

Endothelial cells and progenitor cells migrate following a chemotactic and mechanotactic stimuli to a right place for covering injured portion of a blood vessel or forming new conduits. This highly dynamic process involves complex extracellular matrix-cell and cell-cell interaction and includes chemical sensing of a signal gradient, breaking up intercellular junctions, degradation of extracellular matrix, protrusion of lamellipodia, and cytoskeletal remodeling [42]. There is solid evidence that angiogenic factors like VEGF or angiopoietin-1 utilities ROS for signal transduction and directing cell migration [43–45]. 

**Figure 1:**
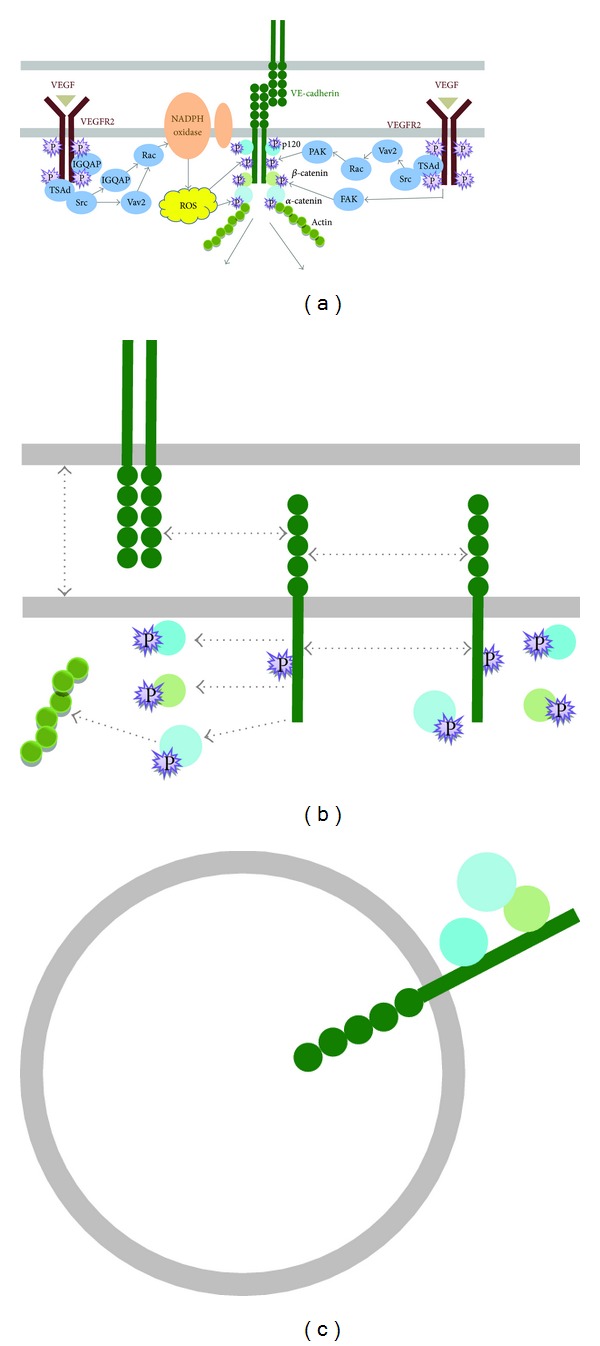
Regulation of intercellular VE-cadherin disruption between endothelial cells by reactive oxygen species and VEGF signaling during EC migration. In basal state, clustering of VE-cadherins between endothelial cells mediate intercellular adhesions. VE-cadherin forms a dimer and bind directly to p120 and *β*-catenin, with the latter associated with *α*-catenin to bridge the actin cytoskeleton. Upon VEGF stimulation, TSAd-dependent Src activation recruits IQGAP1, a multifunctional scaffold protein, to assist association of Rac1 with other Nox subunits (a). Subsequent ROS production by NOx phosphorylate VE-cadherin and *α*-catenins, leading to disassembly of VE-cadherin-catenin complex and EC junctional breakdown, which in turn results in EC migration (b). On the other hand, *β*-catenin phosphorylation by VEGF-induced FAK activation and p120 phosphorylation by thrombin-activated PKC-*α* also promotes the breakdown of endothelial cell tight junctions. Moreover, phosphorylation of VE-cadherin in the cytoplasmic tail via VEGF-VEFGR-Src-Vav2-Rac-PAK axis promotes *β*-arrestin2 dependent of its internalization and disassembly (c).

At the very beginning of migration, quiescent endothelial cells lined in parent vessels need to break up their intercellular connections, of which the major adhesion component is vascular-endothelial- (VE-) cadherin [46]. VE-cadherin forms a dimer and bind directly to *β*-catenin (alternatively to plakoglobin) and to p120, with the latter two also binding to *α*-catenin to link the actin cytoskeleton. A scaffold protein called IQGAP1, which binds to actin, *β*-catenin, CLIP-170, Rac, Cdc42, Calmodulin, and many other cytoskeleton-associated proteins [47], can associate with VE-cadherin and VEGFR in a quiescent endothelial cell [43, 48]. Upon VEGF stimulation, IQGAP1 binds more avidly to activated VEGFR, at same time recruiting Rac1 and Nox subunits to initiate ROS production [43, 49]. Bridging IQGAP1 to VEGFR is further assisted with T-cell-specific adaptor- (TSAd-) dependent activation of c-Src kinase [50, 51], which in turn phosphorylates IQGAP1 [51] and enhances ROS production probably via recruiting more Nox subunits [52, 53] or activating a Rac1-guanine nucleotide exchange factor (GEF) Vav2 [54]. Cysteine sulfenic acid formation in IQGAP by locally produced ROS may also share a role [55]. ROS-dependent phosphorylation of VE-cadherin and catenins leads to disassembly of VE-cadherin-catenin complex and EC junctional breakdown [48, 56–58]. Beta-catenin can be directly phosphorylated by VEGF-induced FAK activation [59], while p120 phosphorylated by thrombin-activated PKC-*α* [60], all facilitating adherent junctions dissociation and ultimately promoting EC migration. Interestingly, phosphorylation in the cytoplasmic tail of VE-cadherin via VEGF-VEFGR-Src-Vav2-Rac-PAK axis promotes *β*-arrestin2 dependent of its internalization and disassembly of intercellular junctions [61], which in turn promoted EC migration (see Figure 1). 

Migrating cells create focal complexes transiently in leading edges and constantly reorganize cytoskeletons to form filopodia or lamellipoda. Localized production of ROS is essential for their function at precise subcellular compartments. A paradigm used by migrating endothelial cells is to tether Nox subunits by different scaffolds or adaptors to different substructures [62]: IQGAP links Nox2 to actin meshwork at the leading edge [49]; WAVE1 recruits p47phox and binds to Rac1 and Rac1 effector PAK, producing ROS and forming membrane ruffles [63]; TRAF4 associate with focal contact scaffold Hic5 as well as p47phox, promoting p47phox-TRAF-Hic complex formation and PAK1-dependent ROS production at focal complexes [64]. A novel protein Poldip2 in VSMC can associate with p22phox to activate Nox4 and RhoA, thus strengthening focal adhesions and stress fiber to promote cell migration [65]. Even cancers take advantage of this strategy to breed podosomes during invasion. In colon cancer cells, p47phox-related adaptor protein tyrosine kinase substrate (Tks) 4 and Tks5 recruit p22phox and facilitate Rac- and Nox1-dependent ROS generation at invadopodia [66, 67]. Thus, compartmentalization of redox signaling is essential for the highly dynamic feature of a moving cell. 

### 4.2. EC Proliferation and Survival

Proliferating endothelial cells generate higher level of superoxide and hydrogen peroxide than in quiescent cells [68]. ROS produced by Nox2 and Nox4 enhances EC proliferation and survival through activation of receptor tyrosine kinases and phosphorylation of p38, ERK, and Akt [5, 68, 69]. In endothelial cells, Nox2 silencing induces activation of apoptotic marker caspase 3/7 [5], while Nox4 overexpression inhibits their activity during serum deprivation [69], suggesting ROS derived from both Nox isoforms exert antiapoptotic effects. 

Under stress condition such as energy deprivation, cells initiate a prosurvival mechanism that degrades damaged cytoplasmic components in lysosomes and recycles new building blocks for renovation, a process known as autophagy [70]. Reactive oxygen species have long been reported to be a signaling mediator of autophagy [71] and to increase endothelial cell survival in response to cell stress [72]. Inhibition of mitochondrial ROS production decreases AMP-activated protein kinase (AMPK) activation, which is involved in chemerin- or 2-Deoxy-D-glucose- (2-DG-) induced endothelial autophagy [72, 73]. Moreover, ROS-mediated autophagy is critical for EC migration and tube formation during angiogenesis [73, 74]. The molecular mechanisms by which ROS regulates autophagy are at least partially due to direct inactivation of a cysteine protease, Atg4, at the site of autophagosome formation, thereby promoting lipidation of Atg8 for autophagosome processing [75]. Excessive oxidative stress, on the other hand, promotes cell apoptosis by activating the death-related pathway, known as type II programmed cell death (PCD). In persistent pulmonary hypertension (PPHN), autophagy of the pulmonary artery endothelial cells (PAECs) is proapoptotic and forms a positive feedback loop with Nox-derived ROS [76]. 

## 5. ROS, VEGF Signaling, and HIF Activation in Angiogenesis

### 5.1. VEGF Signaling

Multiple signaling pathways are activated during angiogenic process by various factors like VEGF, PDGF, angiopoietin, Notch, Wnt, TGF-*β*, and GPCR agonists, with VEGF as a dominating player. VEGF exerts its action through binding to VEGF Receptor-2 (VEGFR-2, also known as FLK1/KDR) in ECs, causing the latter autophosphorylated in its cytoplasmic tyrosine residues and driving downstream pathway such as PI3K/AKT and MAPK to promote EC proliferation and migration. VEGF stimulates ROS production via Rac-1-mediated NADPH oxidase activation [41, 43] and also increases mitochondria-derived H_2_O_2_ [77]. ROS, in turn, potentiate VEGFR phosphorylation [41] and is required for downstream cSrc, FAK, PI3K, and ERK signaling [78]. ROS can also upregulate VEGF secretion and VEGFR expression through induction of transcription factors HIF-1 [79–81].

**Figure 2:**
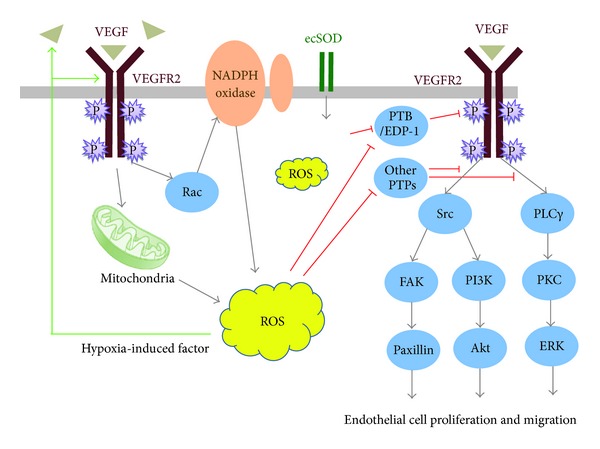
ROS in VEGF signaling in endothelial cell proliferation and migration. Binding of VEGF to VEGFR2 stimulates ROS production via Rac-1 mediated NADPH oxidase activation and through increased mitochondria activity. ROS oxidize and inactivate protein tyrosine phosphatases (PTPs), disinhibiting their negative regulation on downstream signaling pathways, such as Src/PI3K/Akt and PLC/PKC/Raf/ERK. H_2_O_2_ are also generated extracellularly by ecSOD to locally inactivate DEP1 and PTP1B, two PTPs that dephosphorylate VEGF receptor, thereby promoting VEGF-induced VEGFR2 autophosphorylation. Through induction of transcription factors HIF-1, ROS can also upregulate VEGF secretion and VEGFR expression.

The role of ROS in modulating signaling attributes largely to reversible oxidative inactivation of protein tyrosin phosphatase (PTP), which inhibits signaling by dephosphorylating pathway components [82, 83], including the receptor itself [33]. For VEGFR2, PTP1B and density-enhanced phosphatase-1(DEP-1)/CD148 are the major negative phosphatases, and can be inactivated locally in caveolin-enriched lipid rafts by H_2_O_2_ generated by extracelluar superoxide dismutase (ecSOD), and thus facilitating VEGFR2 signaling [84]. In addition, growth factor-activated Src kinase can not only stimulated NAPDH for ROS production, but also phosphorylate and inactivate ROS degrading enzyme peroxiredoxin (Prx1), building up a local H_2_O_2_ gradient to inactivate neighboring protein tyrosine phosphatase and sustain tyrosine receptor signaling [85]. Ultimately, such VEGF-ROS signal pathways promote EC migration and proliferation (Figure 2).

### 5.2. Hypoxia-Induced Factor

Hypoxia, a well-known nonchemical signal for angiogenesis in vascular development and pathological state, also harnesses redox modulating to regulate its responder, hypoxia-induced factor (HIF). HIFs belong to PER-ARNT-SIM (PAS) family of basic helix-loop-helix (b-HLH) transcription factors and have three members: HIF-1, -2, and -3. HIF is a heterodimer composed of an oxygen sensitive HIF*α* subunits and a constitutively stable HIF*β* subunit. Under normal oxygen, HIF*α* is hydroxylated in its proline residues by prolyl hydroxylate proteins (PHDs), thereby generating a binding site for the von Hippel-Lindau (VHL) tumor suppressor protein, which initiates ubiquitin proteasome pathway for HIF*α* degradation [86]. 

**Figure 3:**
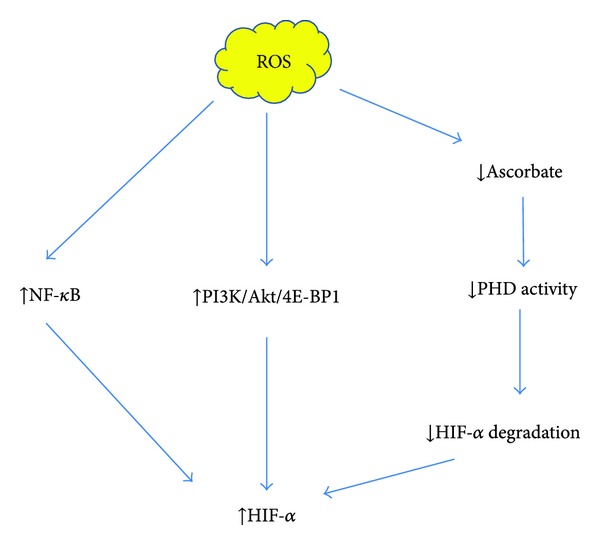
Regulation of hypoxia-induced factor by intracellular reactive oxygen species. Reactive oxygen species positively regulates HIF through enhanced HIF production via activation of NF-*κ*B and PI3K/AKt/4E-BP1 pathway. Meanwhile, ROS inhibits HIF degradation by depleting cellular ascorbate, a cofactor for PHD activity, thus inhibiting HIF*α* hydroxylation and VHL binding.

Angiogenesis induced by urotensin-II, a potent vasoactive peptide, involves feed-forward enhancement between HIF protein and Nox2 [87]. A rapid increase in nox2-derived ROS in response to urotensin stimulation elevates HIF-1*α* level, leading more binding of HIF-1*α* to Nox2 promoter. Nox2 transcription is then enhanced and more ROS are generated to activate HIF-1 further, thus maintaining a positive feedback loop for angiogenesis. In another study, ROS produced by Nox4 in cardiomyocyte can stabilize HIF-1*α* and promote VEGF release to increase myocardial angiogenesis in overload stress [81]. Under hypoxic condition, Nox expression can be readily induced by HIF, participating in cell migration and proliferation. Though this is observed only in pulmonary artery smooth muscle cells, there's reason to expect a similar role in endothelial cells for angiogenesis. How intracellular ROS enhance or stabilize HIF has recently been uncovered. On the one hand, ROS mediate transcriptional activation via NF-*κ*B [88] and translational activation via PI3K/AKt/4E-BP1 pathway [89], increasing HIF production. On the other hand, ROS deplete cellular ascorbate, a cofactor for PHD activity, and inhibit HIF*α* hydroxylation and VHL binding [81, 90, 91], suppressing HIF degradation (see Figure 3). Increased HIF activity promotes angiogenesis.

## 6. ROS and SMC Function Involved in Vascular Formation

Vascular smooth muscle cells, as an important component of blood vessels, function to contract or relax vessel, to regulate blood pressure and distribute blood flow. Smooth muscle cells display striking plasticity and can undergo phenotype switch, dedifferentiating from a quiescent contractile state to a highly migratory synthetic state, in response to vascular injury or various disease states [92, 93]. In this section, we discuss how reactive oxygen species regulate SMC differentiation, proliferation, and migration.

### 6.1. SMC Differentiation

Nascent VSMCs originate from diverge source during mammalian vascular development, including neural crest, proepicardium, mesothelium, secondary heart field, smites, and mesoangioblasts [94]. In injured vasculatures, stem/progenitor cells give rise to smooth muscle cells to form neointima during vascular repair [95]. ES cells can differentiate into SMC in response to growth factors (e.g., PDGF and TGF-*β*), mechanical forces, and certain extracellular matrix (i.e., collagen IV) [96, 97] by activating various signal pathways or gene programme such as integrins-PDGFR *β* crosstalk [96], histone deacetylase 7 [98], transcription factor Sp1 [99], nuclear proteins chromobox protein homolog 3 [100], and heterogeneous nuclear ribonucleoprotein A2/B1 [101]. Importantly, during SMC differentiation and phenotypic modulation, ROS mediated by Nox4, Nrf3, Pla2g7, or other regulators also plays a fundamental role [12–14, 102].

**Figure 4:**
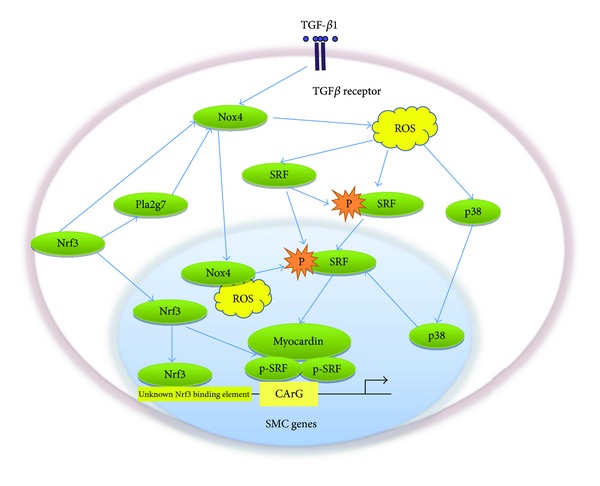
Regulation of SMC differentiation by Nox4-derived ROS. Activated Nox4 by TGF-*β* via its receptor and/or upregulated Nox4 by Nrf3 and/or Pla2g7 leads to up-regulation and phosphorylation of SRF through ROS. The phosphorylated SRF in the nucleus bind to the CArG recruiting myocardin to the promoter of SMC-specific genes. Meanwhile, Nox4-derived ROS can also activate SMC gene expression via p38MAPK pathway. During differentiation, Nox translocates into nucleus. Moreover, Nrf3 and Pla2g7 promote the recruitment of myocardin/SRF complex to CArG box in the promoter region of SMC genes. Importantly, Nrf3 has been shown to bind directly to the unknown Nrf3 binding element within promoter regions of SMC genes.

TGF-*β* is a prodifferentiation factor for smooth muscle cells. It activates Nox4 during SMC differentiation from ES cells [13]. Nox4-derived ROS upregulates the expression and phosphorylation of serum response element (SRF) and drives SRF to translocate into nucleus for SMC gene transcription [13]. In addition, Nox4 expression is enhanced by nuclear factor erythroid2-related factor3 (Nrf3) [12], a member of the cap “N” collar family of transcription factors. Nrf3 can recruit myocardin/SRF complex to CArG box in the promoter region of SMC-specific genes and directly bind to SM*α*A and SM22*α* promoter. Our study also demonstrated for the first time that the fine-tuning of Nrf3-Pla2g7- (phospholipase A2-, group VII) Nox4-ROS axis plays a crucial role in SMC differentiation from ES cells *in vitro* and *in vivo* [14], firmly confirming its functional importance of ROS signals in SMC differentiation and development (see Figure 4).

As stated above, VSMCs can exhibit extensive phenotypic diversity and plasticity and are modulated by numerous environmental cues including growth factors and cytokine, inflammatory cell mediators and lipids. Maintenance of differentiated or contractile VSMCs phenotype can be enhanced by PDGF, TGF-*β*, MMPs, and reactive oxygen species [92]. Nox4 is necessary for smooth muscle markers expression and contractile type stress fibers in VSMCs, through SRF phosphorylation and gene transactivation via p38 MAPK pathway [103, 104]. Notably, the changing of Nox4 localization from stress fibers in differentiated VSMCs to focal adhesions in proliferate cells [103] is reminiscent of the Nox4 translocation into nucleus during SMC differentiation [13]. The subcellular shifting of Nox4 during different cellular state underscores the importance of compartmentalized ROS signaling for specific function [62].

### 6.2. SMC Proliferation

During normal vascular formation and pathological conditions like hypertension and restenosis, vascular SMCs undergo a phenotypic switch to a migratory or proliferative phenotype in response to a variety of growth factors and inflammatory mediators' stimulations. These factors, including PDGF [105], Ang II [106], urokinase plasminogen activator [107], heme [108], urotensin II [109], TGF-*β* [110], and thyroid hormone [111], can activate Nox and subsequent ROS production, promoting smooth muscle cell proliferation. The growth-related downstream signaling pathways are varied among different Noxs isoforms and different stimuli. For example, PDGF-induced SMC proliferation mediated by Nox5 involves JAK/STAT pathway [105], while Ang II stimulation leads to p38 and Akt activation through Nox1 in hypertrophic response [112]. 

### 6.3. SMC Migration

Migration of smooth cells to cover the preexisting collateral arteriolar network is an essential step in arteriogenesis, and provides mechanical support and contractility for a mature blood vessel. The driving forces for the process include fluid shear stress and growth factors such as PDGF, FGF, and TGF-*β*. Since cell migration share many similarities and we have already discussed the case of ECs, here we only summarize some common feature and highlight unique aspects in how ROS influence VSMC migration. 

**Figure 5:**
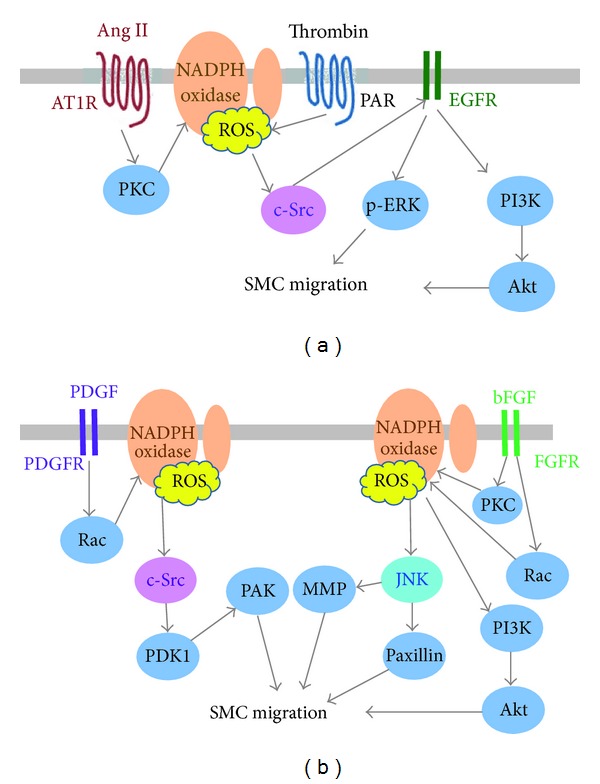
Signaling pathways in ROS-mediated SMC migration activated by agonists. Various agonists such as AngII, PDGF and thrombin activate c-Src in a ROS-dependent way. Signaling cascades downstream of c-Src include EGFR transactivation followed by PI3K/AKT or ERK activation (for AngII) (a) and direct PDK1/PAK phosphorylation (for PDGF) (b). Basic fibroblast growth factor (bFGF) activates PKC and Rac instead of c-Src in smooth muscle cells, but the ultimate JNK or PI3K/Akt activation still requires Nox-derived ROS.

First, certain signaling components controlling migration are modulated by ROS, though which the specific pathway can be different. For example, c-Src activation by various agonists such as AngII, PDGF, and thrombin, is ROS-dependent [113–116]. This important signal node has direct impact on downstream cascades like c-Src-PDK1-PAK [114] or c-Src-EGFR-PI3K/ERK [113, 116], all affecting cell motility. Basic fibroblast growth factor (bFGF), however, activates PKC and PI3K/Akt instead of c-Src in smooth muscle cells, but the ultimate JNK activation still requires Nox-derived ROS [117] (see Figure 5). 

Second, migration depends on degradation of extracellular matrix and loss of cell-matrix and cell-cell adhesion. This often needs the cleavage activity of metalloproteinase (MMP). Similar to the role of ROS in downregulating VE-cadherin in endothelia cells, N-cadherin shedding in disrupting intercellular junction between VSMCs also involves ROS. By Nox1-dependent transactivation of epidermal growth factor receptor, pro-MMP-9 is activated to cleave N-cadherin to promote SMC migration [116]. Another potentially important MMP subtype produced by SMC is MMP2, which can be induced with transcription factor FoxO3a by urotensin-II. Urotensin drive Nox4-dependent activation of JNK and subsequent phosphorylative inactivation of sequestering protein 14-3-3, thereby allowing FoxO3 into the nucleus to enhance MMP2 transcription [109]. In pathological states like hypertension and acute coronary syndrome, increased MMP release by VSMC may link to abnormal extracellular matrix reorganization, deranged VSMC migration and plaque rupture. This, however, can also be mediated though Nox-derived ROS [118, 119]. 

**Figure 6:**
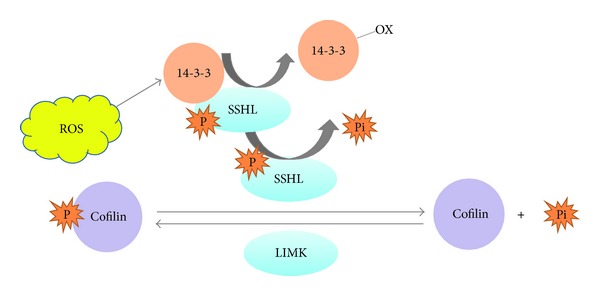
Regulation of slingshot-cofilin pathway by reactive oxygen specious in SMCs. Cofilin is phosphorylated and inactivated by LIM kinase, and p-cofiln is dephosphorylated and activated by phosphatase slingshots-1L. SSHL is sequestered by a regulatory protein 14-3-3. Increasing level of ROS oxidizes 14-3-3, releasing SSH1L phosphatase, which, on the one hand, dephosphorylates other SSH1L molecules to increase SSH1L phosphatase activity. On the other hand, SSH1L dephosphorylates cofilin, which recycles actin monomers for cytoskeletal remodeling during smooth muscle cell migration.

Thirdly, in migrating cells, constant reorganization of cell protrusions (filopodia, lamellipoda, stress fiber, and focal complexes) and cytoskeletons are modulated by ROS, indirectly through ROS-dependent activation of downstream effector kinases, small GTPase and cytoskeleton-associated proteins. Moreover, for contractile cells like VSMCs, contraction regime is another significant target for ROS to modulate. Nox1^y/-^ VSMCs present decreased expression of mDia1, a RhoA adaptor protein, and decreased phosphorylation of cofilin, a regulator of actin depolymerization [120]. Cofilin servers to increase the turnover of actin filaments and is essential for maintaining and protruding lamellipodia. Cofilin is phosphorylated and inactivated by LIM kinase (LIMK), and p-cofiln can be dephosphorylated and activated by phosphatase Slingshots-1L (SSH-1L) [121], which is sequestered by a regulatory protein 14-3-3. ROS produced by Nox1 oxidize 14-3-3, thus releasing SSH-1L to activate cofilin and subsequent cytoskeletal remodeling for migration [122, 123] (see Figure 6). Furthermore, ROS increase intracellular Ca^2+^ mobilization partially through Ca^2+^ influx, thereby enhance VSMC contraction [124]. 

## 7. Perspective

With years of efforts, ROS is becoming increasingly recognized as key modulator for a variety of biological functions and pathophysiological states. Recent evidence across species suggests an even more general and significant role of ROS, including germ line specification in maize [125], root proliferation/differentiation transition in *Arabidopsis* [126], and wound detection in zebrafish [127]. We have discussed above how ROS regulates vascular development in different aspects, including stem cells and SMC differentiation, angiogenesis, VEGF signaling, endothelia progenitor cells recruitment, and vascular cell migration. Nonetheless, much more details regarding the ROS signaling and pathophysiological functions remain to be learn, for example, how the levels of ROS are balanced not to damage biomolecule but to modify normal signal; how ROS are specified and confined, and how ROS in the nucleus modify epigenetic change. Importantly, different forms of ROS like H_2_O_2_ and O^2-^ may display opposing effects. Further studies are needed to clarify their respective action, and how transition between different ROS is coordinated by cells to achieve a specific function. More sensitive and specific tools are also needed for detection and visualization of different ROS species. 

ROS have long been deemed as noxious molecules in cardiovascular disease, including systemic and pulmonary hypertension, atherosclerosis, cardiac hypertrophy, and heart failure. However, some very recent gene knockout and overexpression studies on Nox4 suggest that Nox4-derived ROS have vascular protective function [81]. Thus, the regulation and function of ROS system seem even more complex and intriguing than we previously thought. A better understanding of how different physiological/pathophysiological state would impact on vascular system may resolve the paradox [128–130]. Lastly, deeper insights into the mechanism of how ROS affect normal vascular development, especially SMC and EC differentiation from stem cells, will promise a more bright future on regeneration medicine for cardiovascular therapy.
